# Autosomal recessive inherited bleeding disorders in Pakistan: a cross-sectional study from selected regions

**DOI:** 10.1186/s13023-017-0620-6

**Published:** 2017-04-07

**Authors:** Arshi Naz, Muhammad Younus Jamal, Samina Amanat, Ikram Din ujjan, Akber Najmuddin, Humayun Patel, Fazle Raziq, Nisar Ahmed, Ayisha Imran, Tahir Sultan Shamsi

**Affiliations:** 1 Department of Thrombosis and Hemostasis, National Institute of Blood Diseases and Bone Marrow Transplantation, ST 2/A, Block 17, Gulshan-e-Iqbal, KDA Scheme 24, Karachi, Pakistan; 2 Department of Hematology, Pakistan Atomic Energy Commission Hospital, Islamabad, Pakistan; 3 Department of Pathology, Liaquat University of Medical and Health Sciences, Jamshoro, Pakistan; 4 Fatimid Foundation, Karachi, Pakistan; 5 Department of Hematology, Hayatabad Medical Complex and Lady Reading Hospital, Peshawar, Pakistan; 6 Department of Hematology, Children’s Hospital, Lahore, Pakistan; 7 Department of Hematology, Chughtai’s Laboratory, Lahore, Pakistan

**Keywords:** Autosomal recessive, Inherited bleeding disorders, Coagulation factors, von Willebrand disease type 3, Glanzmann’s thrombasthenia, Bernard–Soulier syndrome

## Abstract

**Background:**

Autosomal recessive bleeding disorders (ARBDs) include deficiencies of clotting factors I, II, V, VII, X, XI, XIII, vitamin K dependent clotting factors, combined factor V & VIII, Von Willebrand Disease (vWD) type 3, Glanzmann’s thrombasthenia (GT) and Bernard–Soulier syndrome. Patients with primary bleeding disorders from all the major provincial capitals of Pakistan were screened for ARBDs. Prothrombin (PT), activated partial thromboplastin time (APTT), bleeding time (BT) and fibrinogen levels were measured. Cases with isolated prolonged APTT were tested for factors VIII and IX using factor assays This was followed by FXI:C level assessment in cases with normal FVIII and FIX levels. vWD was screened in patients with low FVIII levels. Factors II, V and X were tested in patients with simultaneous prolongation of PT and APTT. Peripheral blood film examination and platelet aggregation studies were performed to assess platelet disorders. Urea clot solubility testing was done to detect Factor XIII levels where platelet function tests were normal. Descriptive analysis was done using SPSS version 16.

**Results:**

Of the 429 suspected bleeding disorder patients, 148 (35%) were diagnosed with hemophilia A and 211 (49.1%) patients had ARBDs. 70 patients (16.3%) remained undiagnosed. Out of 211 patients with ARBD; 95 (33.8%) had vWD type 3. Fibrinogen deficiency was found in 34 patients (12%), GT in 27 (9.6%), factor XIII deficiency in 13 (4.6%), factor VII deficiency in 12 (4.3%), factor V deficiency in 9 (3.2%). Eight patients (2.8%) had vitamin K-dependent clotting factor deficiency, Bernard–Soulier syndrome was diagnosed in seven patients (2.5%), factor X deficiency in 2 (0.7%), factor II deficiency in 2 (0.7%), factor XI deficiency and combined factor V and VIII deficiency in 1 (0.4%) patient each.

**Conclusion:**

vWD type 3 was the most common ARBD found in our sample of patients in Pakistan, followed by fibrinogen deficiency and GT in respective order.

## Background

The incidence of autosomal recessive bleeding disorders (ARBDs) worldwide is uncommon at about 3–5% [1, 2] as compared with other causes of bleeding. However, these disorders predominate in those regions of the world where consanguineous marriages are encouraged [3]. Pakistan has a high rate of such marriages [4, 5]. The prevalence of some of these disorders in the local population has only been reported in a few studies [6–11] and a lack of diagnostic facilities and expertise has prevented a comprehensive study to identify ARBDs.

The ARBDs include deficiencies of clotting factors I, II, V, VII, X, XI, XIII, vitamin K-dependent clotting factors [VKDCF; II, VII, IX and X], combined factors V and VIII, von Willebrand disease type 3 (vWD), Glanzmann’s thrombasthenia (GT) and Bernard–Soulier syndrome (BSS). The presentation and bleeding pattern in these patients varies according to the etiology of each disorder [12, 13]. Life threatening bleeding episodes e.g., central nervous system or musculoskeletal bleeding, occur rarely.

Fibrinogen deficiency has a prevalence of 1 in a million [14, 15]. It is subdivided into two distinct phenotypes: quantitative defect (afibrinogenemia and hypofibrinogenemia) and qualitative defect (dysfibrinogenemia and hypodysfibrinogenemia), Prothrombin deficiency (PD) has a prevalence of approximately 1 in two million [16] and has two phenotypes: true hypoprothrombinemia (type I deficiency) and dysprothrombinemia (type II deficiency) [16]. Factor V [FV] deficiency is manifested by skin and mucus membrane bleeding, epistaxis and menorrhagia. Prevalence is 1 in a million [17]. Factor VII deficiency presents as a hemophilia-like bleeding disorder with an estimated prevalence of 1 in 300,000–500,000 [18]. The most severe form of vWD is type 3, characterized by a bleeding disorder associated with a total or near-total absence of von Willebrand factor (vWF) with deficiency of plasmatic factor VIII (FVIII) [8]. The type 3 vW disease is the rarest form of vWD, accounting for less than 5% of all cases of bleeding disorders worldwide. Annual incidence ranges from 1 in two million to 1 in 350,000 in Europe and the United States, with estimates of around 1 per 500,000 in countries where consanguinity is more frequent [19]. Combined deficiency of factor V and VIII is associated with mutations in the LMAN1 and MCFD2 genes [20, 21]. It is characterized by concomitantly low levels (usually between 5 and 20%) of both FV and FVIII and is associated with a mild to moderate bleeding tendency [22]. There are two variants to vitamin K-dependent clotting factor deficiency VKDCF; VKDCF1, associated with point mutations in the gamma-glutamyl carboxylase gene (GGCX), and VKDCF2, which results from point mutations in the vitamin K epoxide reductase gene (VKORC1) [23]. Factor X deficiency has an estimated prevalence of 1 in one million individuals [24]. Factor XI deficiency can manifest first as a bleeding disorder or as an incidental laboratory abnormality. Occurrence is approximately 1 per one million [25]. Factor XIII deficiency is a rare disorder, causing a severe bleeding tendency. The incidence is 1 per one million to 1 in five million people [26, 27]. GT is the most frequently diagnosed inherited disorder of platelet function (prevalence, 1 in a million) [28]. Patients lack or have nonfunctional alpha 2b beta 3 (αIIbβ3) integrin. Type I individuals have <5% of αIIbβ3, while type II have between 10 and 20%. In type III, there are normal levels of αIIbβ3, but they are not functional [29]. The autosomal recessive disorder BSS has a prevalence of 1 in one million [30]. Platelets from patients with BSS lack the major surface membrane glycoprotein complex, glycoprotein (GP) Ib-IX-V [31].

The aim of this study was to determine and compare the 12 year period prevalence of ARBDs in several regions of Pakistan.

## Methods

The study was approved by the ethics committee of the National Institute of Blood Diseases and Bone Marrow Transplantation (NIBD), Karachi, Pakistan, in accordance with the declaration of Helsinki. It was a descriptive study with cross-sectional time prospect, conducted from March 2010 till December 2014.

**Fig. 1:**
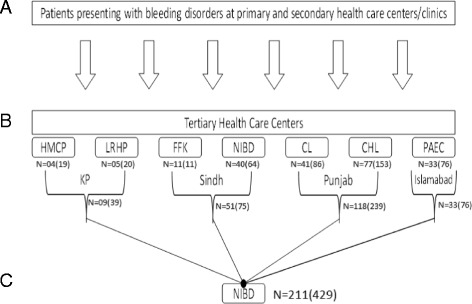
Flow diagram of patient recruitment, sample collection and its disposition at different levels of health care facilities/laboratories. **a** Initial presentation of patients with bleeding disorders at primary and/or secondary health care centers. **b** Referral of patients to the tertiary health care centers or laboratories for definitive diagnosis. Patients were recruited into the study at this point (**c**). Samples sent over to the central reference laboratory NIBD where the tests were repeated to establish reliability. CHL, Children Hospital Lahore; CL, Chughtai’s Laboratory; FFK, Fatimid Foundation Karachi; HMC, Hayatabad Medical Complex; KP, Khyber Pakhtunkhwa; LRH, Lady Reading Hospital; NIBD, National Institute of Blood Diseases; PAEC, Pakistan Atomic Energy Commission. N = number of patients with ARBD, () indicate total number of patients recruited from each center initially

In local set-up, patients are usually diagnosed to have a bleeding disorder at primary and secondary health care centers or general clinics. The confirmatory investigations usually include only the platelets count, bleeding time (BT), Prothrombin time (PT) and activated partial thromboplastin time (APTT). Such cases are hence labelled as merely the bleeding disorder patients. These centers and clinics were asked to refer all their patients with bleeding disorders, both classified and unclassified, to the designated tertiary care centers. The tertiary health care centers included NIBD and Fatimid Foundation Karachi [FFK] at the province of Sindh, Chughtai’s Laboratory and the Children’s Hospital Lahore [CHL] at Punjab. Pakistan Atomic Energy Commission Hospital [PAEC] at the federal capital, Islamabad, and Hayatabad Medical Complex [HMC] and Lady Reading Hospital [LRH]) Peshawar at the province of Khyber Pakhtunkhwa [KPK] (Fig. 1). At their visit, patients were enrolled into the current study after acquiring informed written consents. All the non-classified bleeding disorder cases were included into the study. Those categorized as haemophilia A were also included so as to exclude vWD. Patients taking non-steroidal anti-inflammatory drugs (NSAIDS), steroids, clotting factors or those who had had a platelet transfusion 2 weeks prior to the start of the study were excluded.

A general questionnaire, with basic demographic details, clinical and family histories, and the Tosetto bleeding score questionnaire were filled out for each patient by a doctor at the corresponding recruitment center [32]. The doctors were trained before the commencement of the study with administration of the questionnaires.

8.1 ml venous blood was then collected in three sodium citrate (0.109 M, 3.2%) containing sample collection tubes, each one 2.7 ml in volume. BT, PT, APTT, Factor VIII and IX assays, were determined at the tertiary healthcare centers. In patients with normal platelet count and normal clotting times of PT and APTT, platelet aggregation studies were performed. Peripheral blood samples were analyzed to identify any platelet morphological abnormalities. The platelet aggregation studies were performed on Helena Aggram platelet aggregometer (Helena laboratory, Beaumont Texas, USA) using standard aggregation reagents (ADP, 2.25 μM; adrenaline, 5 μM; collagen, 4 μg/ml; ristocetin, 1.5 mg/ml; arachidonic acid, 500 μg/ml).

An aliquot of the platelet poor plasma, for each patient, was transported to NIBD, the central laboratory, under controlled refrigeration (Fig. 1). Here, the first-line coagulation profile, including PT and APTT were repeated on all the samples, using recombinant tissue factor (Stago, Asnières sur Seine, France). The international normalization ratio (INR) was calculated from the PT using the thromboplastin international sensitivity index (ISI) and the mean normal PT. Fibrinogen levels were measured by Clauss method [33]. Samples with isolated prolonged APTT were further tested for FVIII and factor IX (FIX) using the one stage APTT-based factor assay [34]. If the FVIII and FIX levels were normal then FXI levels were measured (FXI:C). In cases with prolonged PT and APTT, factors II, V and X were tested using an assay based on PT using patient platelet poor plasma, glyoxaline buffer, standard or reference plasma, thromboplastin and calcium. Patients with low levels of FVIII were tested for vWF antigen and vWF ristocetin cofactor. A urea clot solubility test was performed using commercially available thrombin on those patients not diagnosed by other coagulation tests and suspected of having factor XIII deficiency.

To identify the period prevalence for various ARBDs, record from the current study was merged with that from all the studies reported in the last 12 years. For this purpose, the common national (PakMedinet) and international (Pubmed, Google Scholar, ISI Web of science, EMBASE and SCOPUS) databases were screened for studies on ARBDs in Pakistani population.

## Results and Discussion

The study cohort consisted of 429 patients, 250 males and 179 females, with a male to female ratio of 1.3:1. The median age of patients was 11 ± 5 years. A history of consanguinity was present in 89% of cases. Of the 429 patients with diagnosed and suspected bleeding disorders, 211 [49.1%] were diagnosed to have an ARBD, 116 of these were males and 95 females. Among 95 females, 58 were adult. A majority of patients (*n* = 148; 34.49%) had hemophilia A while 70 patients remained undiagnosed. Majority of the ARBD patients had VWD type 3 (Table 2).

The most common symptoms reported by the cohort of patients included gum bleeding (57%), and easy bruising (39%). Spontaneous epistaxis and gum bleeding were found in 6%, whereas menorrhagia was reported in 19% of the adult female patients.

Anemia was found in 48% of the patients. Life-threatening intracranial hemorrhage affected 4% of the patients. Phenotypic presentation of ARBDs is detailed in Table 1.

**Table 1:** Frequency and severity of bleeding

ARBD (N)	Gum Bleeding	Hemarthrosis	Hematoma	Epistaxis	Menorrhagia^c^ x/y	Umbilical Cord Bleed	Traumatic Bleeding	Bruises	ICH	BG^a^	BS^b^
VWD-3 (95)	51	8	15	31	11/19	10	-	12	-	II	13.5
Fib. Def. (34)	2	6	17	9	-	-	23	15	-	II	12.5
GT (27)	23	-	4	19	8/9	16	27	19	-	II	11
FXIII Def. (13)	11	-	6	9	6/6	11	5	9	1	II	11.5
FVII Def. (12)	9	8	7	11	6/9	-	11	9	2	III	12
FV Def. (9)	7	2	6	5	5/7	-	4	5	1	II	11
Vit K Def. (8)	7	-	5	2	3/3	5	7	5	-	II	12
BSS (7)	7	-	7	7	-	-	7	7	-	II	11
FX Def. (2)	1	2	2	-	2/3	2	-		-	II	12
FII Def. (2)	2	1	2	1	1/2	-	-	1	-	II	10
FXI Def. (1)	1	-	1	1	-	-	1	1	-	II	10
FV & FVIII Def. (1)	1	1	1	-	-	-	-	1	-	II	11.5

*ARBD* autosomal recessive bleeding disorders, *BG* bleeding grade, *BS* bleeding score, *BSS* Bernard Soulier syndrome, *Def.* deficiency, *Fib.* fibrinogen, *FII* Factor II, *FV* Factor V, *FVII* Factor VII, *FX* Factor X, *FVIII* Factor VIII, *FXI* Factor XI, *FXIII* Factor XIII, *GT* Glanzmann Thrombasthenia, *ICH* intracranial hemorrhage, *N* number of patients, *No.* number, *Vit* vitamin, *VWD-3* von Willebrand Disease type 3, *x* affected females, *y* females at risk

^a^Calculated on the basis of WHO bleeding grades

^b^based on Tossetto et al bleeding score calculation scale

^c^Reported in adult female patients only

Gum bleeding was more prominent in patients with Glanzmann Thromboasthenia and Bernard-Soulier syndrome; hemarthrosis was most common in patients suffering from factor VII deficiency, hematoma was more noticeable in patients with factor XIII, factor V and vitamin K dependent clotting factor deficiency. Patients with factor XIII deficiency had the highest incidence of prolonged umbilical cord bleeding. Prolonged bleeding after trauma was associated with factor VII and vitamin K dependent clotting factor deficiency, GT and BSS. Easy bruising is a prominent feature of GT, BSS and factor XI deficiency according to our study cohort (Table 1).

In the current study, 32 patients were found to have severe fibrinogen deficiency while two patients had moderate severity of the disease. In FXIII deficiency, all the cases had severe disease. Among FVII deficient patients, 2 had mild, 8 had moderate and 2 patients had severe disease. Of the nine patients with FV deficiency, 7 had moderate and 2 had severe disease. All the cases with FX deficiency had severe disease. Two patients had FII deficiency, both bearing moderate severity of the disease. One patient with severe FXI deficiency was also identified. Mild Combined FV & FVIII deficiency was also found in a single case, as per laboratory phenotype classification [13].

**Fig. 2:**
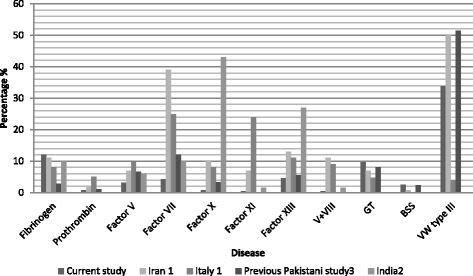
Comparative studies of different nationalities with ARBDs [7, 29, 36]

Findings from the current study were compared with those conducted in Italy [35], Iran [35] and India [36], countries with high rates of autosomal recessive diseases due to consanguineous marriages (Fig. 2). In our study patients, vWD type 3 was the most common disorder, with 95 patients (33.8%), although in a similar, local study, the percentage was 51.4% [7], in Iran it was 50% [35], and in Italy, just 4% [35]. It is hence concluded that in south Asian population vWD type 3 has a high frequency among ARBDs. The second most common deficiency found in this study was fibrinogen deficiency (*n* = 34, 12%). The disease was found to have a frequency of 11% in the Iranian study [35], 8% in the Italian study [35] and 10% in the Indian study [36]. Our study findings are comparable to those from the mentioned contemporary studies. GT, a relatively well understood platelet disorder, was diagnosed in 27 (9.6%) of patients. Its frequency was 6.9% in the Iranian study, 4.7% in the Italian study, and 8.1% in an earlier Pakistani study (Fig. 2).

South Asian countries, particularly Pakistan, have a high frequency of consanguineous partnerships [4], which explains the increased prevalence of ARBD in this region. The local 12 years period prevalence of ARBDs [37, 38] compared with the international prevalence is shown in Table 2. Data was not available from the Baluchistan, Gilgit-Baltistan and Azad Kashmir regions. A larger national study is needed to cover the underprivileged, difficult to access areas of Pakistan, not included in the current study due to the poorly structured healthcare system and difficulties with law and order in these regions. Worldwide data has clearly shown that there is variation in the prevalence of individual ARBDs. Genetic studies to identify the underlying mutations would help in understanding the phenotype/genotype relationship.

**Table 2:** Frequency of ARBDs from different provinces of Pakistan

ARBD	Sindh	Punjab	Federal capital	KPK	Total	Percentage	Previously reported cases from Pakistan^¶^	Total	Local prevalence~ Per million	International prevalence* Per million
VWD type 3 disorder	05	62	21	7	95	(33.8%)	61	156	1.0	0.5
Fibrinogen deficiency	11	20	3	0	34	(12%)	9	43	0.3	0.5
Glanzmann Thrombasthenia	18	9	0	0	27	(9.6%)	50	77	0.5	1
Factor XIII deficiency	7	2	4	0	13	(4.6%)	29	42	0.3	0.5
Factor VII deficiency	4	6	1	1	12	(4.3%)	84	96	0.6	2
Factor V deficiency	0	9	0	0	9	(3.2%)	28	37	0.2	1
Vitamin K dependent clotting factors deficiency	0	7	0	1	8	(2.8%)	0	8	0.04	1
Bernard Soulier syndrome	3	0	4	0	7	(2.5%)	5	12	0.07	1
Factor X deficiency	1	1	0	0	2	(0.7%)	41	43	0.3	1
Factor II deficiency	0	2	0	0	2	(0.7%)	10	12	0.07	0.5
Factor XI deficiency	1	0	0	0	1	(0.4%)	1	2	0.01	1~
Combined Factor V & VIII deficiency	1	0	0	0	1	(0.4%)	0	1	0.006	1

There were no patients from the province of Baluchistan, Gilgit Baltistan and Azad Jammu & Kashmir due to lack of health and diagnostic facilities

*International prevalence data from world hemophilia database and orphanet journal of rare diseases

~Frequency is 1 in 450 in Ashkenazi Jews

^¶^[6, 37, 38]

~Period Prevalence calculations were based on CDC formulation

There is also a need to educate the general population regarding the risks of ARBDs and to initiate genetic counseling services to help prevent consanguineous marriage in families with a history of these disorders. Patients with ARBDs require lifelong management and education on lifestyle modifications relevant to the bleeding disorder that they live with.

## Conclusion

These data have shown that vWD type 3 has the highest incidence amongst the ARBDs in this study cohort, followed by fibrinogen deficiency. GT was found to be the third most common disorder. The incidence of ARBDs in this region is higher than previously thought.

## Abbreviations

APTT: Activated partial thromboplastin time
ARBDs: Autosomal recessive bleeding disorders
BT: Bleeding time
CFD: Clotting factor deficiency
GT: Glanzmann’s thrombasthenia
NIBD: National Institute of Blood Diseases and Bone Marrow Transplantation
PFD: Platelet functional disorder
PT: Prothrombin time
VKCFD: Vitamin K-dependent clotting factor deficiency
vWD: von Willebrand disease
